# Case Report: Functional characterization of lymphocyte populations in a pediatric patient with WHIM syndrome

**DOI:** 10.3389/fimmu.2026.1847018

**Published:** 2026-06-29

**Authors:** Marialaura Mastrovito, Fatima Al-Naimi, Maria Carla Giarratana, Gianluca Dell’Orso, Damiano Lemmi, Federica Raggi, Katarina Zmajkovicova, Lars Karlsson, Sandra Zehentmeier, Maurizio Miano

**Affiliations:** 1 X4 Pharmaceuticals (Austria) GmbH, Vienna, Austria; 2 Haematology Unit, IRCCS Istituto Giannina Gaslini, Genova, Italy

**Keywords:** WHIM syndrome, primary immunodeficiency, CXCR4 gain-of-function, lymphocyte function, neutropenia, myelokathexis, case report

## Abstract

WHIM syndrome is a rare primary immunodeficiency disorder caused by gain-of-function mutations of the chemokine receptor CXCR4, leading to abnormal and exacerbated leukocyte trafficking. It is associated with severe neutropenia and lymphopenia, and recurrent infections. Few pediatric cases have been reported, but comprehensive analyses of the composition and function of peripheral lymphocyte populations in pediatric WHIM syndrome are lacking. Here we report the case of a 6-year-old male patient carrying a *CXCR4* c.1000C>T gain-of-function mutation, presenting with characteristic manifestations such as abnormal orientation of the cerebellar folia, myelokathexis and severe neutropenia as well as lymphopenia. In-depth analysis of peripheral lymphocyte subpopulations revealed an elevated CD4/CD8 T-cell ratio while T-cell activation, expansion and cytokine production were normal. B lymphopenia was accompanied with a shift in circulating B-cell populations towards transitional stages, and increased apoptosis. Interestingly, despite normal serum immunoglobulin levels, vaccination responses and B-cell receptor dependent activation, the patient’s B cells showed a reduced capacity for plasmablast differentiation *in vitro*. These findings warrant follow-up studies in larger patient cohorts, as longitudinal monitoring of B-cell function might reveal emerging humoral defects in WHIM pediatric patients reaching adolescence and adulthood.

## 1 Introduction

WHIM syndrome (warts, hypogammaglobulinemia, infections, and myelokathexis) is a rare autosomal dominant inborn error of immunity, associated with dysregulated leukocyte trafficking including abnormal bone marrow retention of neutrophils and other leukocytes. It is predominantly caused by gain-of-function (GOF) variants of C-X-C chemokine receptor 4 (CXCR4) leading to impaired receptor internalization and hyperactive signaling. WHIM syndrome is characterized by severe neutropenia, lymphopenia, and highly variable multisystem manifestations such as increased risk of severe and/or recurrent infections, malignancy, and subsequent disease complications (1–4). While some of the clinical phenotypes in WHIM syndrome can be attributed to lymphopenia and compromised function of the adaptive immune system (5), datasets on T and B-cell function in patients with WHIM syndrome are scarce. We report the case of a 6-year-old male patient referred for leuko-neutropenia (white blood cell count (WBC) 1.02 x 10^9^/L, absolute neutrophil count (ANC) 0.06 x 10^9^/L), absolute lymphocyte count (ALC) (0.11 x 10^9^/L) and lymphadenopathy unresponsive to oral antibiotic and anti-inflammatory therapy. Next-generation sequencing revealed a “*de novo*” c.1000C>T GOF mutation of the *CXCR4* gene located on chromosome 2q22, suggesting the diagnosis of WHIM syndrome. This case report not only provides a detailed clinical description of a pediatric case of WHIM syndrome, but a comprehensive phenotypical and functional characterization of the patient’s lymphocyte populations.

## 2 Case description

### 2.1 Main clinical findings

**Figure 1:**
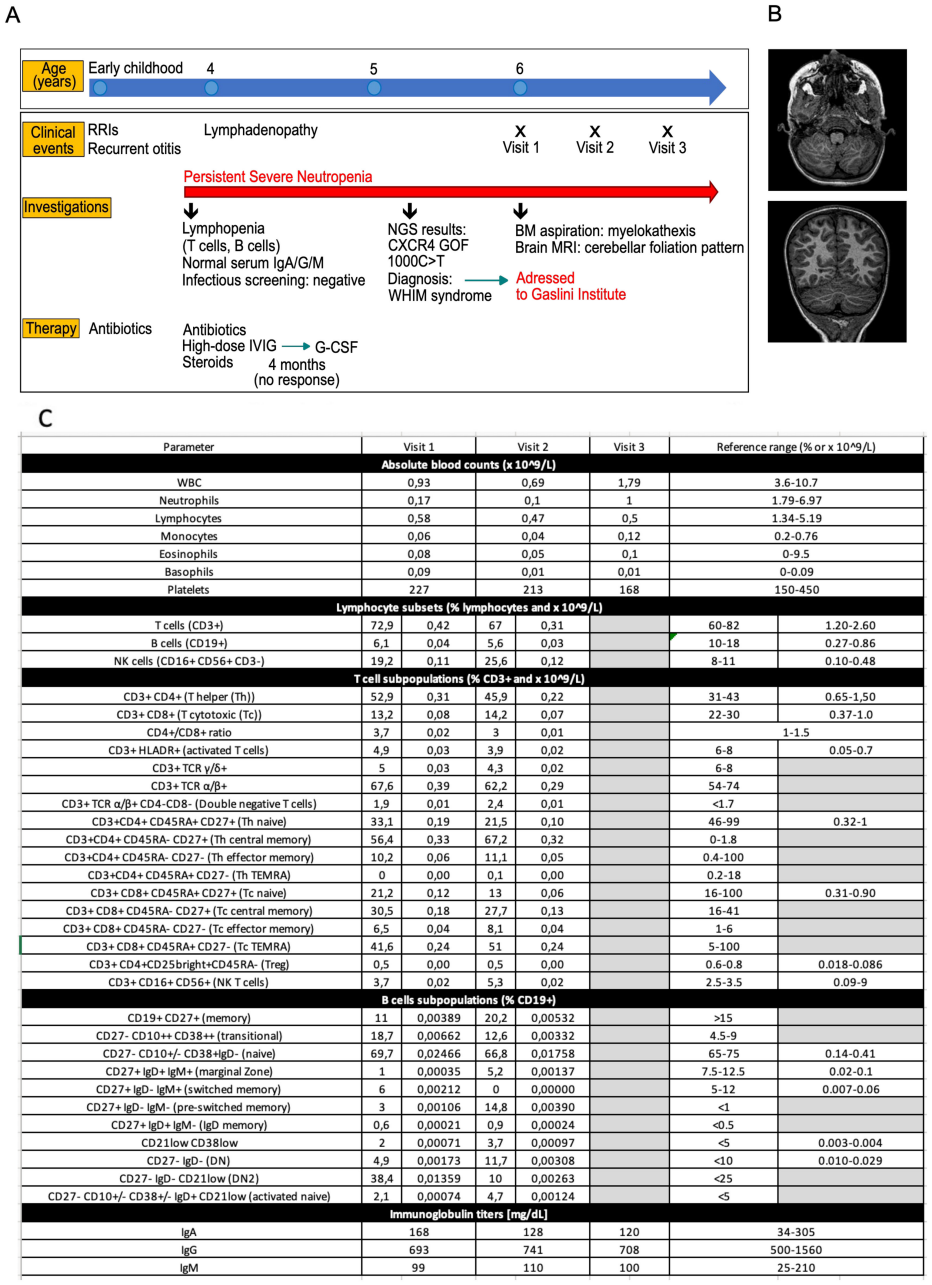
Timeline of clinical events, MRI results and selected blood parameters. **(A)** Timeline of clinical events. RRIs, recurrent respiratory infections; NGS, next generation sequencing; BM, bone marrow; MRI, magnetic resonance imaging; IVIG, intravenous immunoglobulin; G-CSF, granulocyte colony-stimulating factor; **(B)** MRI images **(C)** The patient’s absolute blood counts (x10^9^/L), frequencies of lymphocyte subsets (%) and immunoglobulin titers (mg/dL) are shown for 2–3 consecutive visits (7, 32, 33).

The patient’s family history was notable only for a mother with multiple sclerosis but lacked a formal diagnosis of primary immunodeficiency. The patient was born at term. No physical abnormalities were detected at birth except for a left side asymptomatic inguinal wart, that was in differential diagnosis with a linear verrucous epidermal nevus and monitored overtime without warning signs. Since early childhood, he had a history of respiratory tract infections and recurrent acute otitis media, promptly responsive to oral antibiotic therapy until the age of 4 years (**Figure 1A**). He was regularly vaccinated and thrived normally. At the age of 4 years, he presented with lymphadenopathy associated with leuko-neutropenia and lymphopenia. For this reason, the patient was originally evaluated in another medical center where markers of viral infection (antibodies against Epstein-Barr virus, human cytomegalovirus, human immunodeficiency virus, human herpesvirus 6, hepatitis C virus, parvovirus B19, adenovirus), *Leishmania* (antibodies) and tuberculosis infection (blood tuberculosis test) were negative. Liver and pancreatic function including fecal pancreatic elastase were normal. Autoimmunity screening (anti-nuclear, anti-extractable nuclear antigen, anti-transglutaminase, anti-thyroglobulin, and anti-thyroid peroxidase antibodies) was negative. Peripheral blood lymphocyte subpopulation phenotyping revealed a consistently low CD4^+^ T-cell, CD8^+^ T-cell and CD19^+^ B-cell count. Despite lymphopenia, serum immunoglobulin titers (IgM, IgA, IgG) and IgG subclass levels were normal at diagnosis and during the whole follow-up. Vaccination responses were appropriate for diphtheria, tetanus, mumps, rubella, varicella and pneumococcus. The molecular screening detected a “*de novo*” *CXCR4* c.1000C>T (p.Arg334*) GOF mutation supporting the diagnosis of WHIM syndrome. The mutation was not detected in his parents. A bone marrow evaluation revealed the hallmark feature of myelokathexis. Subsequently, the patient was treated with intravenous antibiotics, intravenous immunoglobulin (IVIG) and steroid therapy without any clinical improvement. Due to persistent severe neutropenia the patient was treated with granulocyte-colony stimulating factor (G-CSF), which maintained the ANC above 0.5 x 10^9^/L and led to the reduction of the adenopathy and progressive clinical improvement. G-CSF injections at a dose of 10µg/kg/dose each other day maintained neutrophil count between of 0.5–2.0 x 10^9^/L for four additional months and were then interrupted.

At the age of 6 the patient was referred to our center (**Figure 1A**). To determine the presence of characteristic multisystem manifestations previously reported for WHIM syndrome, a complete disease-specific diagnostic work-up was performed. Brain magnetic resonance imaging (MRI) showed an abnormal orientation of the cerebellar folia typically described in WHIM patients (**Figure 1B**) (6). Hearing loss was ruled out and cardiac evaluation showed no congenital conotruncal heart defects. Chest CT scan was negative for lung lesions and abdominal ultrasound showed no organomegaly. Complete blood count and serum immunoglobulin values were determined on first presentation at our center (**Figure 1C**, Visit 1). Due to the lymphopenia, anti-pneumocystis prophylaxis was started to reduce risk for opportunistic infection, and anti-human papillomavirus (HPV) vaccination was recommended. To date, the patient is alive and well without treatments. CXCR4 inhibitor has never been administered as it is off-label for age and the patient did not experience any relevant infection.

### 2.2 Peripheral lymphocyte counts, subsets, and serum immunoglobulin titers

We investigated peripheral lymphocyte numbers and serum immunoglobulins on three separate visits within a period of 7 months (**Figure 1C**; **Supplementary Experimental Procedures**). Besides neutropenia and monocytopenia, the patient presented with lymphopenia at all visits with an ALC ranging between 0.47 – 0.58 x 10^9^/L. Peripheral blood B- and T-lymphocyte subsets were determined by diagnostic immunophenotyping for two visits. The patient displayed a consistently low CD19^+^ B-cell frequency, elevated frequency of transitional B-cells (CD27^-^CD10^++^CD38^++^) and low frequency of marginal zone B-cells (CD27^+^ IgD^+^ IgM^+^) compared to the healthy reference range for this age group (7). The memory B-cell compartment was shifted towards pre-switched memory cells (CD27^+^ IgD^-^ IgM^+^), with a normal switched memory B cell (CD27^+^ IgD^-^ IgM^-^) frequency on the first visit, but undetectable levels of switched memory B cells thereafter, coinciding with severe lymphopenia. T-cell findings were consistent for both visits. We observed a ≈ 3-fold elevated CD4/CD8 T-cell ratio as previously reported for patients with WHIM syndrome (8). The CD4^+^ T-cell compartment was shifted towards central memory cells (> 30-fold elevated over the healthy reference) with a reduction of naїve T-cell frequency. CD8^+^ effector memory frequency was above, while CD4^+^ TEMRA and regulatory T-cell frequency were below the healthy range. Interestingly, double negative (DN) T cells were above the healthy range on both visits. The frequency of NK cells was about twofold elevated. Serum immunoglobulin titers (IgM, IgA, IgG) were normal on all visits.

### 2.3 T-cell function

**Figure 2:**
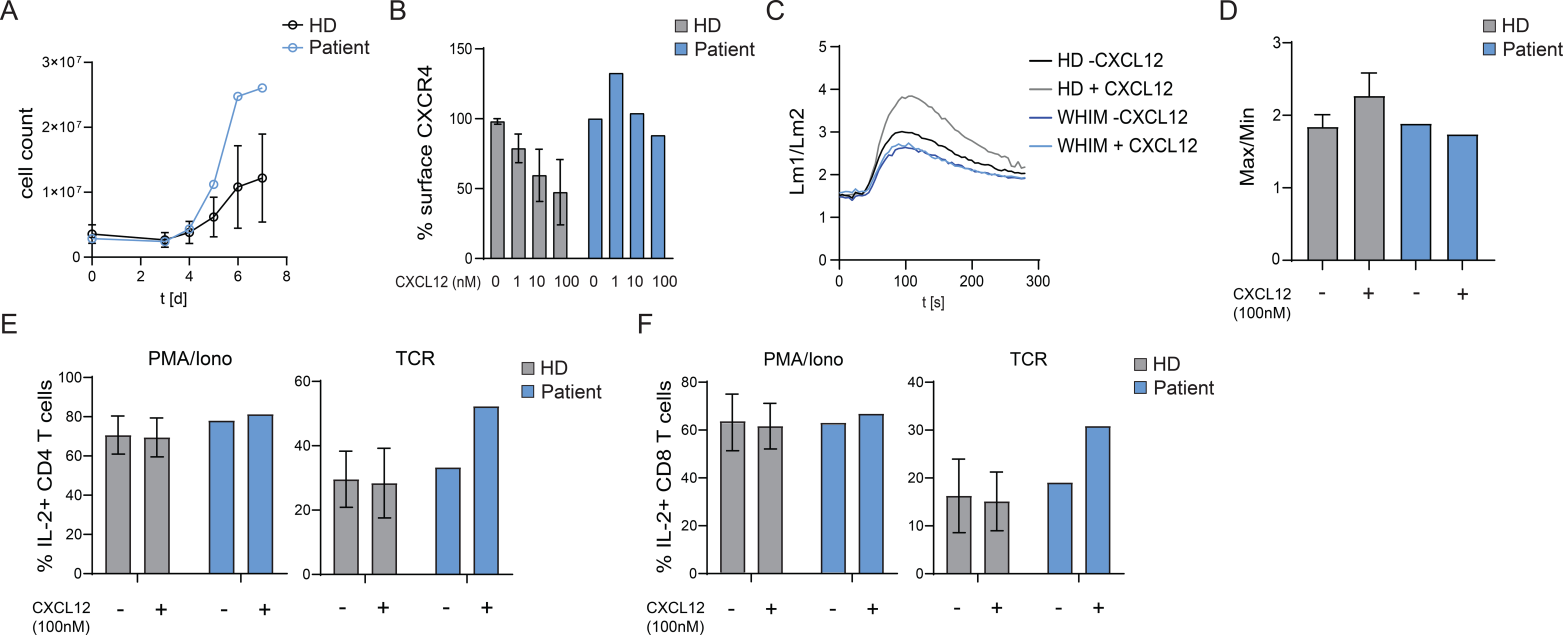
No general impairment of activation induced expansion, TCR activation or cytokine production in expanded patient T cells. **(A)** *In vitro* expansion of PBMC-derived T cells after TCR activation and rhIL-2 exposure, displayed as cell count for patient vs. HD controls. Circles show mean +/- SD. HD n=9 and patient n=1. **(B)** CXCR4 internalization assay on expanded T cells showing % of remaining CXCR4 on the cell surface after 45min of CXCL12 incubation; Bars show mean +/- 95% CI. HD n=9 and patient n=1. **(C)** Calcium-flux post-TCR activation measured via Fura-2AM, patient and representative HD profiles **(D)** Quantification of C, displaying maximum value divided by minimum value. Bars show mean +/- 95% CI. HD n=7, patient n=1. **(E)** Frequency of IL-2^+^ cells among expanded CD4^+^ T cells after 4h of restimulation +/- CXCL12.Bars show mean +/- 95% CI. HD n=8 and patient n=1 **(F)** Frequency of IL-2^+^ cells as in **(E)** among expanded CD8^+^ T cells. HD, healthy donor; PBMC, peripheral blood mononuclear cells; CI, confidence interval.

Given the observed shifts in T-cell subpopulation composition, we performed functional analyses of the patient’s peripheral blood T cells after activation-induced expansion *in vitro*. We did not observe impaired but rather increased expansion compared to healthy donor (HD) T cells (**Figure 2A**), while the patient’s T cells exhibited a CXCR4 internalization defect characteristic of most WHIM mutations (**Figure 2B**) (9–12). When investigating calcium flux after TCR engagement, we found TCR activation of the patient’s T cells in the absence of CXCL12 to be comparable to HD cells. If HD T cells were exposed to CXCL12 before TCR activation, calcium flux was enhanced compared to TCR triggering alone. Notably, this costimulatory effect was absent in the patient sample (**Figures 2C, D**). Next, we analyzed the T cells’ ability to produce IL-2 after TCR activation in the presence or absence of CXCL12. We observed comparable frequencies of IL-2+ cells in both patient and HD within CD4 and CD8 compartments (**Figures 2E, F**). Overall, despite showing the typical internalization defect of pathogenic CXCR4 variants, expanded patient T cells were functionally largely intact.

### 2.4 B-cell developmental stages and function

**Figure 3:**
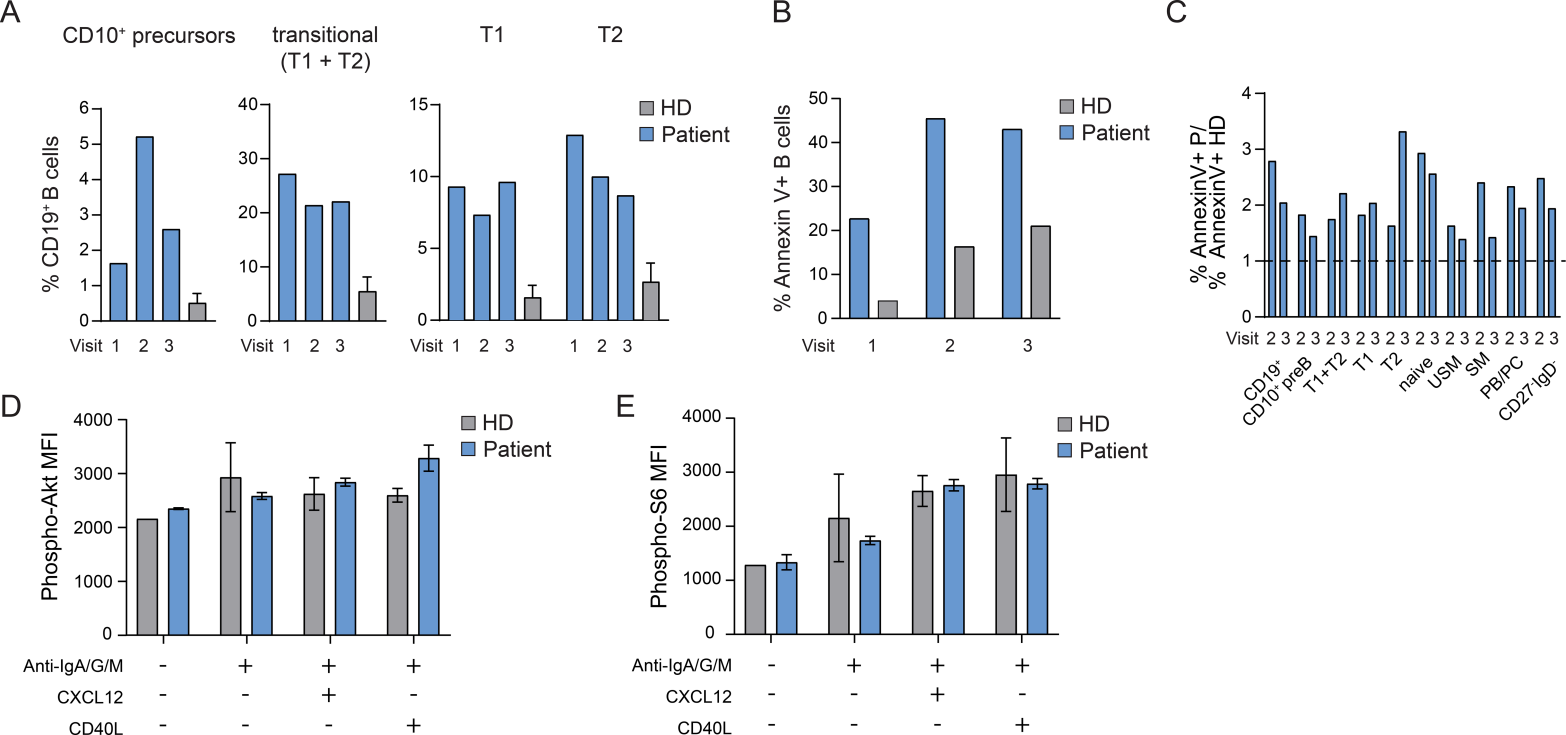
Patient circulating B lymphocytes display elevated frequencies of developing and transitional B cells, and an increased frequency of apoptotic cells but normal B-cell activation *in vitro*. **(A)** The frequency of CD19^+^ CD10^+^ CD38^int^ IgM^-^ IgD^-^ B-cell precursors, T1 and T2 B cells in CD19^+^ B cells of patient PBMC from 3 visits and HD controls was determined by cytometry. Bars show single values (patient) or mean + 95% CI (HD controls, n= 9 individuals). **(B)** Elevated apoptosis in patient samples of 3 visits in total B cells: % of apoptotic CD19^+^ cells as determined as day 0 after thawing of PBMC. Data from 3 patient visits compared to HD. **(C)** The frequency of AnnexinV^+^ cells in B-cell subpopulations of patient PBMC from visit 2 and 3 normalized for HD controls acquired in the same experiment. Bars show single values. **(D, E)** PI3K signaling after B-cell stimulation: MFI of phosphorylated Akt **(D)** and S6 **(E)** in CD19^+^CD27^-^IgM^+^ naїve B cells after 45 minutes stimulation of PBMC samples with anti-IgA/G/M, in presence or absence of CXCL12 and CD40L. Data from one patient visit. Bars show mean +/- SD from n=1–2 technical replicates. Data plotted as geometrical mean. HD, healthy donor; PBMC, peripheral blood mononuclear cells; CI, confidence interval; MFI, mean fluorescence intensity.

As described in section 2.2, despite normal naїve B-cell frequencies in peripheral blood, the patient presented with elevated transitional B cells. Thus, we investigated the composition of the patient’s circulating B-cell compartment at higher resolution and with particular focus on B-cell developmental and transitional stages. Salzer et al. recently reported the presence of a circulating pre B-cell stage population in patients with WHIM syndrome (13, 14). This population, characterized as CD19^+^ CD10^+^ CD38^+^ IgM^-^ IgD^-^ CD21^-^ CD27^-^, appeared independently of patient age and was absent in HD and non-WHIM disease controls (13, 14). Notably, we detected CD19^+^ CD10^+^ CD38^int^ IgM^-^ IgD^-^ cells, and elevated T1 and T2 B-cell frequencies in the patient’s PBMC (**Figure 3A**). Following a previous report of increased apoptosis in B cells from patients with WHIM syndrome concomitant with increased basal activation of WHIM B cells (15), and to investigate whether specific subsets, such as transitional B cells, are enriched for apoptotic cells, we assessed the frequency of apoptotic B cells by Annexin V staining. Of note, the patient’s PBMC showed higher frequencies of apoptotic B cells compared to HD controls (**Figure 3B**). Increased apoptosis of CD19^+^ B cells was consistent on two separate visits across B-cell subsets, with a trend towards a more pronounced apoptosis in naïve B cells (**Figure 3C**). We further assessed the activation status of the patient’s naїve B cells (CD19^+^, CD27^-^, IgM^+^) by evaluating PI3K signaling after B-cell receptor (BCR)-mediated stimulation. Phosphorylation levels of Akt and S6 in patient and healthy B cells were comparable, even in the presence of CXCL12, and CD40L co-stimulation (**Figures 3D, E**), suggesting that the activation of the PI3K pathway in patient B cells was normal.

**Figure 4:**
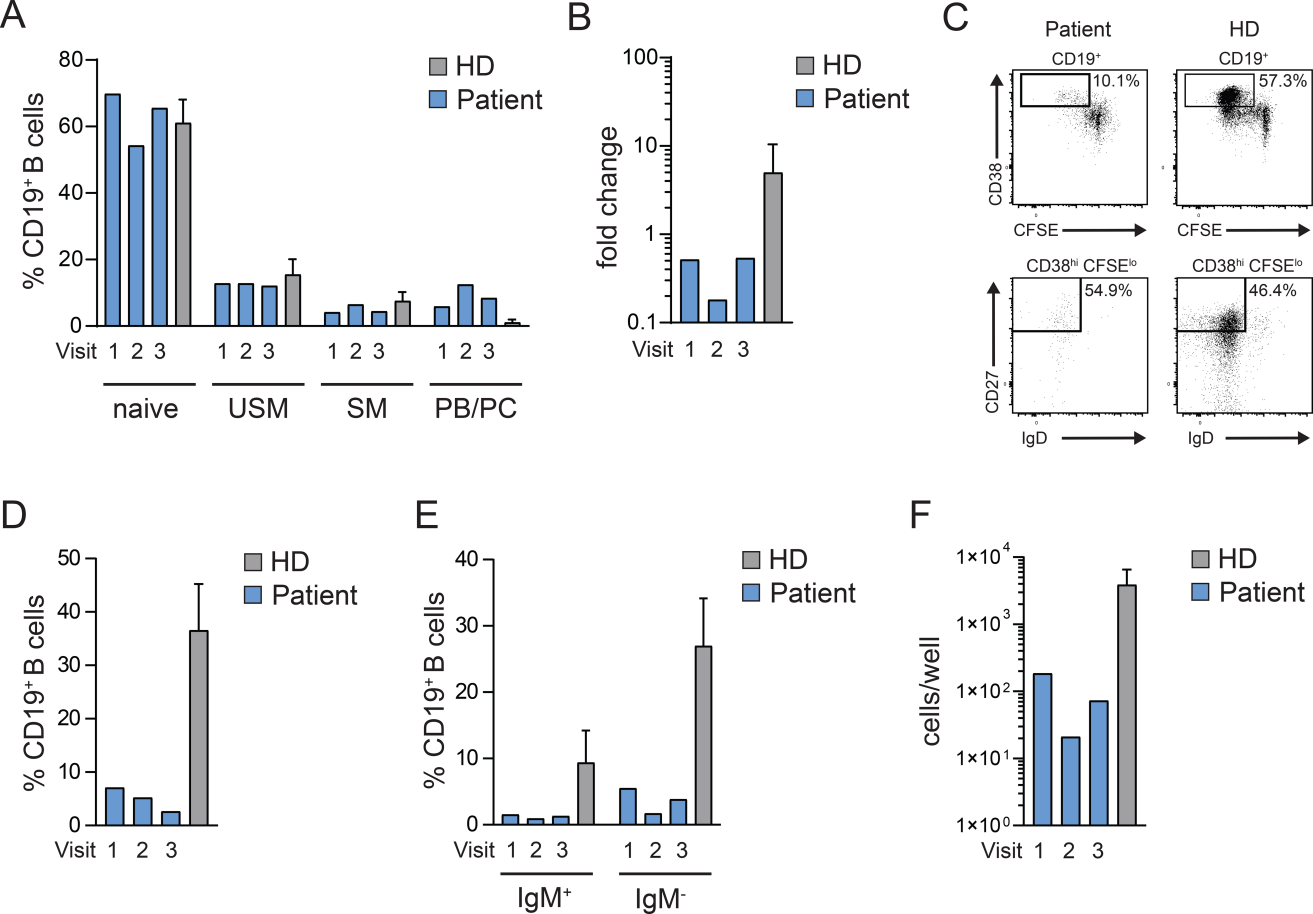
TLR-mediated plasmablast differentiation *in vitro* is impaired in patient PBMC. **(A)** The frequency of naïve B cells, unswitched memory B cells (USM), switched memory B cells (SM) and plasmablasts (PB)/plasma cells (PC) in CD19^+^ B cells of patient PBMC from 3 visits on Day 0 of the culture compared to HD. **(B)** Fold expansion of CD19^+^ B-cell counts on Day 6 of stimulation with R848 and rhIL-2. **(C)** Representative flow cytometry gates for the quantification of PB frequency and counts on Day 6. Displayed events were gated on live, Lin^-^, CD19^+^ cells (top row) and live, Lin^-^, CD19^+^ CD38^hi^ CFSE^lo^ cells (bottom row). PB were identified as CD38^hi^ CFSE^lo^ CD27^hi^ IgD^-^. **(D)** Frequency of PB in CD19^+^ B cells of patient PBMC from 3 visits and HD controls on Day 6. **(E)** Frequency of IgM^+^ PB and IgM^-^ PB in CD19^+^ B cells of patient PBMC from 3 visits and HD controls on Day 6. **(F)** PB counts on Day 6 of the culture of patient PBMC from 3 visits and HD controls. Bars show single values (patient) or mean + 95% CI (HD controls) **(A–E)**. HD data from n= 9 individuals **(A)**, or from n= 8 individuals (displayed as mean values of 1–5 experiments per donor) **(B, D–F)**. HD, healthy donor; PBMC, peripheral blood mononuclear cells; CI, confidence interval.

After interrogating BCR-dependent naïve B-cell activation, we analyzed the potential of patient memory B cells for plasmablast (PB) differentiation by stimulating patient PBMC with the TLR7/8 agonist resiquimod (R-848) in the presence of rhIL-2 *in vitro.* Notably, while the frequency of naїve, unswitched memory (USM, IgD^+^ CD27^+^) and switched memory (SM, IgD^-^ CD27^+^) cells of CD19^+^ B cells in patient PBMC was observed within the 95% confidence interval of a HD cohort for all visits (**Figure 4A**), patient B cells showed reduced expansion after 6 days of stimulation (**Figure 4B**). Consistently, both the frequency and absolute number of CD19^+^ CD38^hi^ CFSE^lo^ CD27^hi^ IgD^-^ PB on day 6 of the culture were reduced, irrespective of IgM surface expression, and fell outside the range defined by a HD cohort (**Figures 4C–F**), indicating a defect of PB differentiation *in vitro*.

## 3 Discussion

We report an in-depth analysis of B and T-cell function for a pediatric case of WHIM syndrome. Information on lymphocyte development, function, and memory compartments in patients with WHIM syndrome remains scarce. Single reports have investigated isolated aspects of either T-cell (16) or B-cell function (15, 17) in patients with WHIM syndrome, but functional analyses of both cellular components of the adaptive immune system in the same patient have not been reported.

Aside from neutropenia and lymphopenia, peripheral blood lymphocyte subpopulation phenotyping revealed characteristic features previously reported for patients with WHIM syndrome patients, such as an elevated CD4/CD8 T-cell ratio (8, 18, 19). A shift in the patient’s peripheral blood T-cell composition in favor of central memory CD4^+^ T cells and effector memory CD8^+^ T cells at the expense of naïve populations are in line with previous reports of WHIM patients in the same age group (18). Based on investigations in WHIM mouse models, the elevated CD4/CD8 T-cell ratio in WHIM syndrome may be explained by increased thymic retention of CD8^+^ T cells (8) and efflux of WHIM CD8^+^ T cells from the circulation to the bone marrow (8, 20). Additionally, dysregulated T-cell function could contribute to shifts in the circulating T-cell subsets. While the patient’s T cells exhibited the defective CXCL12-induced CXCR4 internalization characteristic for patients carrying the *CXCR4* c.1000C>T GOF variant (10), we did not observe major defects in activation induced expansion, in line with a previous report (21). Ca^2+^ flux triggered by TCR activation in the absence of CXCL12 was not affected in the patient’s T cells. Interestingly, we observed a lack of TCR co-stimulation by CXCL12 in patient T cells, in contrast to reports in healthy T cells (22). Overall, the lack of a major T-cell activation and IL-2 production phenotype fits the patient’s clinical presentation, and is consistent with an absence of opportunistic infections characteristic for severely defective T-cell immunity (2). Previous reports from larger WHIM patient cohorts on increased susceptibility to HPV infections (1, 2, 10, 23, 24) and, in some cases, difficulties to clear HSV infections (2) point to subtle defects in T-cell function, not detected by the assays used in this study.

We observed several shifts in the patient’s peripheral B-cell composition, including an increased frequency of transitional B cells. Whether transitional B-cell counts are elevated remains to be determined. Possibly, this relative enrichment of transitional cells in the circulating fraction is the result of a more pronounced loss of circulating naïve B cells. While we observed an increase in the apoptotic fraction across B cell stages, naïve B cells displayed the highest fold-increase in apoptotic frequency compared to HD, which would be in support of this interpretation. It remains to be elucidated whether transitional B-cell differentiation in individuals with WHIM syndrome is affected, as has been reported for WHIM mouse models (20, 25). Interestingly, in the circulating fraction of this patient, we detected a similar B-cell population as previously reported in 13 WHIM patients by Salzer et al.: a circulating B-cell precursor considered unique to patients with WHIM syndrome (13, 14). While this observation might hint at a dysregulation of early B-cell development in human WHIM syndrome, it seems counter-intuitive - as CXCR4 GOF signaling would be expected to increase retention of B cell progenitors in the bone marrow - and warrants additional investigations on the origin of this population. The activation state of B and T cells after antigen encounter is considered a crucial determinant of a functional humoral immune response, resulting in sustained class-switched antibody production and memory B-cell formation. Thus, alternatively or in addition to aberrant trafficking, dysregulated B-cell activation might lead to humoral defects observed in WHIM patients. B-cell activation in the context of WHIM syndrome has been mostly studied using the S338X WHIM mouse model (15, 26, 27) and based on these studies, a link between CXCR4 GOF and elevated BCR signaling through the PI3K/AKT/mTOR pathway was proposed. Human WHIM B-cell activation studies remain scarce. Roselli et al. reported elevated basal levels and a larger fold increase of the activation marker CD69 at 18 hours post stimulation by BCR cross-linking in the presence of CXCL12 for peripheral B cells from two WHIM patients (15). For this pediatric patient, we have directly assessed PI3K/AKT/mTOR pathway activation in B cells and did not detect differences in the activation profile compared to a HD. While this discrepancy could be linked to differences in stimulation time or strength, it highlights the need for further investigations of PI3K/AKT/mTOR signaling in WHIM B cells from larger patient cohorts to understand whether the dysregulated B cell activation reported in WHIM mice can be translated to patients with WHIM syndrome.

Previous studies found frequencies of circulating switched memory B cells reduced in WHIM patients (17, 28). Notably, this patient did not show a consistent reduction of switched memory B-cell frequency. However, while frequencies of CD27^+^ IgD^-^ memory B cells (CD19^+^ CD27^+^ IgD^-^ IgM^+^ plus CD19^+^ CD27^+^ IgD^+^ IgM^-^) were in the healthy range for the patient’s age group as detected by immunophenotyping on two visits (9% and 14.8% on visit 1 and 2, respectively) (7), we detected a shift from fully switched to pre-switched memory cells in the circulating CD27^+^ IgD^-^ memory B-cell subset. Interestingly, the patient did not present with hypogammaglobulinemia, a WHIM feature with varying penetrance (5, 17, 21, 23), and responded properly to vaccinations. Despite the lack of these more pronounced humoral phenotypes, we uncovered functional defects of the patient’s memory B cells *in vitro*. Human memory B cells can be selectively activated by TLR7 or TLR9 agonists to proliferate and differentiate to plasmablasts *in vitro* (29, 30). B-cell expansion and plasmablast differentiation in the patient’s PBMC samples was reduced compared to HDs, equally affecting the differentiation of IgM^+^ and IgM^-^ plasmablasts. Given comparable frequencies of switched memory B cells observed in the PBMC fraction of the patient and HD controls, this phenotype rather seems to be the result of a functional defect than numeric differences in WHIM and HD samples. A reduced reactivity of the patient’s memory B cells to TLR stimulation or a reduced ability of the patient’s B cells to survive in culture are potential mechanisms. The latter would be in line with the elevated frequency of apoptotic B cells in the patient’s sample. Detailed mechanistic studies analyzing memory B-cell generation and re-activation, as well as plasma cell differentiation potential in B cells from WHIM patients, ideally in response to vaccine antigens, will be required to clarify whether this represents a general, so far unrecognized, phenotype and to dissect its underlying mechanism.

Despite the lack of a clear reduction of switched memory B-cell frequency in the patient’s circulating B-cell compartment as reported in other WHIM cases (17, 28), it is important to note that reduced switched memory B cells are not consistently reported in pediatric WHIM patients (31). Similarly, hypogammaglobulinemia has a variable penetrance in WHIM patients, and is rarely diagnosed in early but rather in late childhood or adolescence (1, 18, 30). It remains to be assessed whether the B-cell phenotypes reported in this study such as the presence of unique circulating B-cell precursors or defective PB differentiation *in vitro* could serve as early indicators of humoral defects in WHIM syndrome.

## Data Availability

The original contributions presented in the study are included in the article/**Supplementary Material**. Further inquiries can be directed to the corresponding author.
