# Supplementary material for: Case Report: Functional characterization of lymphocyte populations in a pediatric patient with WHIM syndrome
Source: Front Immunol. 2026 Jun 29;17:1847018. doi: 10.3389/fimmu.2026.1847018 (PMC13357159; doi:10.3389/fimmu.2026.1847018)
Supplement: Supplementary file 1 [file DataSheet1.docx]

**Supplementary Material**

1. **Supplementary Experimental Procedures**

## Peripheral blood mononuclear cell (PBMC) preparation

PBMC were isolated from patient or adult healthy donor whole blood by Ficoll gradient (Histopaque 1077; Sigma-Aldrich, Cat#10771). PBMC were frozen in cryomedium and stored in liquid nitrogen until analysis. Cells were thawed according to standard protocols and incubated in Rosswell Park Memorial Institute (RPMI) 1640 with GlutaMax and HEPES (Gibco, Cat#72400-047) supplemented with 1% Penicillin/Streptomycin (Gibco, Cat#15140122) and 20% FBS (Gibco, Cat#A47622-01) for 1 hour before performing analyses or cell-based assays. Patient samples were tested alongside healthy donor controls in cell-based assays.

## T cell expansion

T cells were expanded from frozen PBMC by culturing them in RPMI1640 with GlutaMax and HEPES (Gibco, Cat#72400-047) supplemented with 1% Penicillin/Streptomycin (Gibco, Cat#15140122)) and 20% FBS (Gibco, Cat#A47622-01)), and activating them with ImmunoCult Human CD3/CD28/CD2 T Cell Activator (StemCell Technologies, Cat#10970) for three days at 37°C 5% CO_2_. On day three, cells were counted and cultured at 1x10^6^/mL in complete medium supplemented with 10 ng/mL rhIL-2 (Peprotech, Cat#200-02-500µg). Expanded T cells were kept at 1x10^6^/mL for up to 7 days in complete medium with rhIL-2. Functional T cell assays were performed on day 5 to day 7 of culture.

## Receptor internalization assay

Cells were adjusted to 1x10^6^/mL in starvation medium (RPMI1640 (Gibco, Cat# 72400-047) + 1% FCS (Gibco, Cat# A47668-01) + 1% Penicillin/Streptomycin) and seeded with 1x10^5^/ well in 200µL. Samples were incubated at 37°C, 5% CO_2_ for 1h. After starvation, cells were briefly spun and supernatant discarded. Then, 200µL CXCL12 (PeproTech, Cat#300-28A-1MG) dilutions (100nM, 10nM and 1nM) were added. Cells were incubated at 37°C, 5% CO_2_ for 45min. Subsequently, samples were washed twice with ice cold incubation buffer (Hanks' Balanced Salt Solution (HBSS) with Ca2^+^ and Mg^2+^ (Gibco, Cat# 14065-049) + 0.5% BSA (Sigma A7030-500G) + 20 mM HEPES (Gibco, Cat# 15630-080, pH 7.4) and Fc receptors were blocked with Human TruStain FcX (BioLegend, Cat#422301) for 10 min on ice. Surface staining was performed on ice for 30min. Washed cells were resuspended in flow buffer (HBSS with Ca2^+^ and Mg^2+^ + 0.1% BSA + 20 mM HEPES pH 7.4) and acquired on CytoFlex S. Antibodies used: anti-CD184 (CXCR4)(BD Pharmigen, 12G5), anti-CD3 (BioLegend, HIT3a).

## Calcium flux assay

Expanded T cells were adjusted to 5x10^6^/mL in calcium mobilization buffer (HBSS (Gibco, Cat# 14065-049), 20mM HEPES (Gibco, Cat# 15630-080), 0,375 g/L NaHCO3 (Fisher-Scientific, Cat# BP357-1), 0,1% BSA(Sigma, A7030-500G)) and labelled with Fura-2 AM: Pluronic F-127 mix (1:1) (Thermo Fisher, Cat# F1221;Invitrogen, Cat# P3000MP) at 4 µM for 30min at 37°C 5% CO_2_. After washing, cells were resuspended at 6x10^6^/mL and plated into black 96-well clear-F-bottom plates (300,000/ well). 50 µL of corresponding CXCL12 dilutions were added and wells filled up to 100 µL with calcium mobilization buffer. After 30 min of pre-incubation with CXCL12, calcium flux was performed at Flexstation (Molecular Devices) with TCR stimulation via CD3e (CD3 Monoclonal Antibody (OKT3), eBioscience, 10 µg/mL) at 20 s followed by a full calcium release at 280s reading time with ionomycin (1 µg/mL). The full read time was 6 min.

## T cell cytokine assay

Cells were adjusted to 1x10^6^/mL and 100,000 cells were taken to be stimulated via a) TCR or b) PMA/Ionomycin for 4 h with Brefeldin A added during the last 2 h. Samples were analyzed on a Cytoflex S flow cytometer (Beckman Coulter), using CytExpert (Beckman Coulter) and FlowJo. live/dead cell staining was done using the LIVE/DEAD Fixable Yellow Dead Cell Stain Kit (Invitrogen, Cat# L34959) and following manufacturer’s instructions. Afterwards, Fc receptors were blocked with Human TruStain FcX for 10 min on ice. Surface staining was performed on ice for 30 min. Samples were washed with flow buffer and fixed, permeabilized and stained intracellularly using the BD Cytofix/Cytoperm Fixation/Permeabilization Kit (BD Bioscience, Cat# 554714) according to manufacturer’s instructions.

The following antibodies were used for staining: anti-CD184 (BD Bioscience, 12G5), anti-CD56 (NCAM) (eBioscience, TULY56), anti-CD45RO (BioLegend, S19021B), anti-CD27 (BioLegend, M-T271), anti-CD197 (CCR7) (BioLegend, G043H7), anti-CD45RA (BioLegend, HI100), anti- IL-2 (BioLegend, MQ1-17H12), anti-CD14 (BioLegend, 63D3), anti-CD19(BioLegend, HIB19), anti- CD4 (BioLegend, RPA-T4), anti-CD8 (BioLegend, SK8), anti- TCR a/b (BioLegend, IP26).

## B cell activation assays

BCR based B cell activation: B cells from a pool of PBMC from the patient and a matching healthy donor control were stimulated using AffiniPure F(ab´)2 Fragment Goat Anti-Human IgA/G/M (10 mg/ml, Jackson ImmunoResearch), in presence or absence of human Mega CD40L (0,1 mg/ml, Enzo Life Sciences , ALX-522-110), human CXCL12 (10 nM) for 45 minutes at 37°C). Plasmablast differentiation culture: For *in vitro* plasmablast differentiation, viable cell counts of PBMC samples were determined manually and the frequency of CD19^+^ B cells PBMC samples was determined by flow cytometry. PBMC were labelled with a final concentration of 0.2µM CellTrace CFSE (Thermo Fisher, Cat# C34554) according to manufacturer’s instructions to track cell division and seeded in RPMI 1640 with GlutaMAX and HEPES containing inactivated 10% fetal bovine serum and penicillin/streptomycin (P/S) at equal numbers of CD19^+^ B cells per well in a 96 well plate. Cells were stimulated with 2.5µg/mL resiquimod (R848) (Sigma, Cat# SM0196-50MG) and 1000U/mL rhIL-2 for 6 days at 37°C. On Day 0 (pre-stimulation) and Day 6 of the culture, PBMC were analyzed for B cell subpopulations including plasmablasts (CD19^+^ CD38^hi^ CFSE^lo^) by flow cytometry. Cell counts per well were determined by flow cytometry, by recording all cells per well at a defined volume.

## Flow cytometry

Single cell suspensions of PBMC were stained with antibodies in HBSS (with Ca2^+^ and Mg^2+^) supplemented with 0.5% bovine serum albumin (BSA), 20mM HEPES, for cell surface staining or in Annexin V Binding Buffer (BioLegend, Cat#422201) for determination of apoptotic cells by Annexin V staining (Alexa Fluor 647 Annexin V, BioLegend, Cat# 640912). Fc receptor mediated Ig Fc binding was blocked with Human TruStain FcX reagent, DAPI (Sigma Aldrich, Cat#9542-5MG) was used for live/dead cell discrimination. For phospho-Akt and pospho-S6 analysis, PBMC were stimulated as indicated above. Cells were fixed using the Intracellular Fixation & Permeabilization Buffer Set (eBioscience, Cat# 88-8824-00) for 20 min on ice and underwent surface staining using the following antibodies: anti-CD19 (BioLegend, HIB19), anti-CD21 (BioLegend, Bu32), anti-CD69 (BioLegend, FN 50), anti-CD148 (BioLegend, A3), anti-IgM (BioLegend, MHM-88), anti-CD27 (BioLegend, M-T271). Cells were then stained intracellularly using anti-S6 (pS235/pS236) antibody (BD Biosciences, N7-548) and anti-Akt (pS473) antibody (BD Biosciences, M89-61) in permeabilization buffer, for 30 min at room temperature.

Stained cells were analyzed in HBSS, 0.1% BSA, 20mM HEPES on a Cytoflex S flow cytometer (Beckman Coulter, Brea, CA, USA). Data were analyzed with FlowJo software (TreeStar, Ashland, OR, USA). For *in vitro* assays, within CD19^+^ B cells (Lineage (CD3, CD14, CD56)^-^ DAPI^-^ CD19^+^), B cell subpopulations were defined as follows: CD10^+^ circulating B cell precursor: CD10^+^ CD38^int^ IgM^-^ IgD^-^; Transitional: IgD^+^ CD38^hi^; T1: IgD^+^ CD38^hi^ CD27^-^ CD10^+^; T2: IgD^+^ CD38^hi^ CD27^-^ CD10^-^; Naïve: IgD^+^ CD27^-^; Unswitched memory (USM): IgD^+^ CD27^+^; Switched memory (SM): IgD^-^ CD27^+^  or IgD^-^ CD27^+^ CD38^-/int^; PB/PC: IgD^+^ CD38^hi^;

Antibodies used in the study are listed in Supplementary Table 1.

## Statistical analysis

For healthy donor controls, data are presented as mean with error bars showing the 95% confidence interval (CI). The number of healthy donor controls analyzed is indicated in the figure legends. Data graphs were plotted using Prism software (GraphPad Software, Boston, MA, USA).

1. **Supplementary Table 1**

Supplementary Table 1: Flow cytometry antibodies used in this study.

| **Antibody** | **Clone** | **Supplier** | **Cat#** | **RRID** | |
| --- | --- | --- | --- | --- | --- |
| CD3e | OKT3 | eBioscience | 14-0037-82 | AB_467057 | |
| OptiBuild Brilliant Violet 750 Mouse anti-human CD184 | 12G5 | BD Bioscience | 747265 | AB_2871981 | |
| APC Mouse IgG2a, κ Isotype Control | G155-178 | BD Pharmigen | 555576 | AB_398604 | |
| anti-human TCR a/b PercP-Cy5.5--- | IP26 | BioLegend | 306724 | AB_2563002 | |
| anti-human IL-2 PE | MQ1-17H12 | BioLegend, | 500307 | AB_315094 | |
| anti-human IgM APC-Cy7 | MHM-88 | BioLegend | 314519 | AB_10897095 | |
| anti-human IgD PE-Cy7 | IA6-2 | BioLegend | 348210 | AB_10680462 | |
| anti-human CXCR4 BV421 | 12G5 | BD Bioscience | 562448 | AB_11153865 | |
| anti-human CD8 FITC | SK1 | BioLegend | 344704 | AB_1877178 | |
| anti-human CD56 (NCAM) eFluor 450 | TULY56 | eBioscienc | 48-0566-42 |  | |
| anti-human CD56 (NCAM) Brilliant Violet 421 | 5.1H11 | BioLegend | 362552 | AB_2566061 | |
| anti-human CD56 (NCAM) Brilliant Violet 605 | TULY56 | eBioscience | 406-0566-42 | AB_3093231 | |
| anti-human CD45RO APC/Fire 750 | S19021B | BioLegend | 376805 | AB_3083367 | |
| anti-human CD45RA PE/Dazzle 594 | HI100 | BioLegend | 304146 | AB_2564079 | |
| anti-human CD4 APC | RPA-T4 | BioLegend | 300537 | AB_2562051 | |
| anti-human CD38 Alexa Fluor 647 | HB-7 | BioLegend | 356632 | AB_2721419 | |
| anti-human CD3 Brilliant Violet 421 | UCHT-1 | BioLegend | 300434 | AB_10962690 | |
| anti-human CD3 Alexa Fluor 488 | HIT3a | BioLegend | 300320 | AB_493691 | |
| anti-human CD3 PE | HIT3a | BioLegend | 300308 | AB_314044 | |
| anti-human CD27 PE-Cy7 | M-T271 | BioLegend | 356412 | AB_2562258 | |
| anti-human CD27 Brilliant Violet 711 | M-T271 | BioLegend | 356429 | AB_2650750 | |
| anti-human CD21 - PerCPCy5.5 | Bu32 | BioLegend | 354908 | AB_2561544 | |
| anti-human CD197 (CCR7) PE | G043H7 | BioLegend | 353204 | AB_10913813 | |
| anti-human CD19 Brilliant Violet 605 | HIB19 | BioLegend | 302244 | AB_2562015 | |
| anti-human CD19 APC-Cy7 | HIB19 | BioLegend | 302218 | AB_314248 | |
| anti-human CD19 Alexa Fluor 488 | HIB19 | BioLegend | 302219 | AB_389313 | |
| anti-human CD19 eFluor 450 | HIB19 | eBioscience | 48-0199-42 |  | |
| anti-human CD184 (CXCR4) PE | 1D9 | BD Pharmigen | 551510 | AB_394228 | |
| anti-Human CD184 (CXCR4) BV421 | 12G5 | BD Pharmigen | 566282 | AB_2739656 | |
| anti-human CD14 Brilliant Violet 421 | ME5E2 | BioLegend | 301830 | AB_10959324 | |
| anti-human CD14 BV605 | 63D3 | BioLegend | 367126 | AB_2716231 | |
| anti-human CD10 Brilliant Violet 510 | HI10a | BioLegend | 312220 | AB_2563835 | |
| anti-human CD69 BV605 | FN50 | BioLegend | 310938 | AB_2562307 | |
| anti-human CD148 PE | A3 | BioLegend | 328708 | AB_2174826 |  |
| AffiniPure F(ab´)2 Fragment Goat Anti-Human IgA/G/M | Polyclonal | Jackson ImmunoResearch | 109-006-064 | AB_2337548 |  |
| anti-human S6 (pS235/pS236) Alexa Fluor 647 | N7-548 | BD Biosciences | 560435 | AB_2869348 |  |
| anti-human Akt (pS473) Alexa Fluor 488 | M89-61 | BD Biosciences | 560404 | AB_1645342 |  |
